# Optimized Calibration of Terahertz Polarimetric Imaging Systems With Imperfect Polarizers for Accurate Jones-Matrix Mapping

**DOI:** 10.21203/rs.3.rs-10271350/v1

**Published:** 2026-07-08

**Authors:** Arash Karimi, Zachery B. Harris, M. Hassan Arbab

**Affiliations:** Department of Biomedical Engineering, Stony Brook University, Stony Brook, NY 11794, USA; Department of Biomedical Engineering, Stony Brook University, Stony Brook, NY 11794, USA; Department of Biomedical Engineering, Stony Brook University, Stony Brook, NY 11794, USA

**Keywords:** Terahertz polarimetry Imaging, polarimetric calibration, wire-grid polarizer, condition number, leaky polarizer, Jones matrix, eigendecomposition

## Abstract

Accurate terahertz (THz) polarimetric imaging requires robust system calibration to recover the Jones matrix of a sample. In many THz systems, calibration procedures often rely on wire-grid polarizers (WGPs) that exhibit frequency-dependent leakage when the wire period is comparable to the operating wavelength, which can introduce systematic errors. In this work, we develop an *in situ* calibration framework for a polarimetric THz scanner in which an imperfect WGP is rotated in front of a mirror placed at the sample position to provide a complete set of calibration measurements. The calibrating WGP is modeled as a lossless but leaky element having a frequency-dependent extinction coefficient and phase retardance. We derive two equivalent calibration formulations, one of which is based on an orthogonal harmonic-basis formulation that enables a Fourier series interpretation and suggests selecting rotation angles that are uniformly spaced modulo π. The condition numbers of the two alternative matrix equations are minimized to optimize the performance of the system with respect to the choice of polarizer rotation angles. Using this approach, calibration can be performed with a minimal set of three optimized angles, yielding a relative error of less than 10% compared to a reference calibration with a full set of 18 angles. We validate the resulting sample characterization procedure using a rotating x-cut lithium niobate crystal, where accounting for WGP leakage removes systematic bias and produces reflection coefficients consistent with the theoretical predictions. Finally, we demonstrate accurate polarimetric imaging by mapping the Jones matrices of two birefringent crystal samples with unknown principal axes via eigendecomposition of calibrated measurement matrix and compared with theoretical values. This optimized calibration technique enables faster and more reliable THz polarimetric imaging in field-deployable scenarios by minimizing the number of required calibration measurements.

## I. Introduction

Terahertz (THz) polarimetry and ellipsometry enable measurement of polarization-dependent responses in complex systems, thereby improving quantitative material characterization [1]–[4]. Applications of THz polarimetry span the study of anisotropic materials, including birefringence, dichroism, and chirality [5]–[10], metamaterials [11]–[14], biological tissues and molecules [15]–[20], composite materials [21]–[23], thin-film structures [24]–[26], and the magnetooptic effect [27]–[30]. In addition, THz polarimetry enables investigation of scattering and depolarization phenomena in rough surfaces [31], granular structures, and turbid media [32]–[34]. Polarimetric THz imaging extends these concepts to spatially resolved measurements by mapping the Jones or Mueller matrix of targets [35]–[38]. In imaging systems that measure multiple polarization bases, an accurate reconstruction of a sample’s Jones matrix requires calibration of the instrument response, including channel gain imbalance and polarization cross-talk introduced by optical components, beam steering, and detection electronics [39]–[41]. These effects are especially relevant in beam steering architectures, where the polarization state of the incident beam can vary across the field of view (FOV) as a function of steering angles and optical geometries [35], [42], [43].

With rapid advances in THz technology, diverse imaging systems have emerged, differing in application, speed, bandwidth, and portability [2], [44]–[46]. We developed the THz Portable HAndheld Spectral Reflection (PHASR) Scanner, based on a fiber-coupled photoconductive antenna (PCA) emitter-detector pair. The system uses a gimbal mirror and a telecentric f-θ lens to steer and focus the beam across the field of view (FOV) [47]. Owing to its portability and high acquisition rate, PHASR Scanner has been used primarily for *in vivo* burn assessment studies [48], [49]. However, skin and other biological samples have granular structures, which can give rise to Mie scattering [32], [33], [50], [51]. Although these scatterers are embedded in a strongly absorbing medium, scattering can still contribute to a measurable secondary signal contrast mechanism [32], [33]. This scattering-based contrast, together with broader needs in applications involving anisotropic structures, motivated the development of a polarimetric version of the THz PHASR Scanner [35].

The polarimetric THz PHASR Scanner, considered in this work, uses a fiber-coupled PCA emitter and two orthogonally oriented detector channels to record the perpendicular components of the electric field. In this architecture, the detected field can be expressed as a product of the sample response and the Jones matrices of the system that capture any change in the polarization state of the propagating beam and the gain response of the detection channels [35]. Therefore, both the incident field at the sample and the system response must be carefully calibrated to recover the sample properties from the measured data. We recently introduced an *in situ* approach to calibrating THz polarimetric systems by placing a rotating wire-grid polarizer (WGP) in front of a mirror at the sample position, producing a known polarization transformation as a function of rotation angle [35]. However, popular WGPs are not ideal optical components in broadband THz frequencies, because when the wire period is comparable to the THz wavelengths the frequency-dependent leakage of the polarization component parallel to the wires is not negligible [52], [53]. Ignoring this leakage in the calibration model can bias the recovered Jones matrix parameters and propagate a systematic error in subsequent sample characterization. In the Supplementary Material, we analyze this error propagation and show that it scales approximately with the square of the WGP extinction coefficient. In addition, because the calibration parameters are estimated by a finite set of angle-dependent measurements, the numerical conditioning of the resulting system of equations can affect how measurement noise and experimental uncertainty propagate into calibration error. Therefore, the system matrices should be analyzed to obtain the optimum rotation angles of the WGP by minimizing the condition number of the calibration equations [54], [55].

In this study, we present an optimized calibration framework that explicitly accounts for imperfect (leaky) polarizers while reducing the measurement burden without sacrificing accuracy. The calibrating WGP is modeled as a lossless leaky element parameterized by an extinction coefficient and phase retardance. We characterize the frequency-dependent leakage of free-standing tungsten WGPs using independent transmission measurements. We then describe an *in situ* calibration method in which the WGP is rotated in front of a reference mirror placed at the scanner’s reflection focal plane. Because the calibration parameters are estimated from a finite set of angle-dependent measurements, the numerical conditioning of the resulting equations governs how measurement noise and experimental uncertainties propagate into the calibration error. We therefore analyze the system matrices and select optimal WGP rotation angles by minimizing the condition number of the calibration equations [54], [55]. Finally, we present two equivalent angle-dependent formulations of the calibration equations. Minimizing the condition number of the corresponding equation system matrices produces two unique optimal angle sets. In one preferred formulation, an orthogonal harmonic-basis approach yields a Fourier-series interpretation in which calibration measurements should be obtained at angles that are uniformly spaced modulo π. In the alternative equation system, this optimization resulted in the unique angle sets of [45°, 113°, 157°] and [23°, 67°, 135°]. This analysis provides a practical guideline: selecting polarizer rotation angles to minimize the condition number of the system matrix reduces error propagation, enabling a minimal calibration using only three angles.

We demonstrate the proposed method on the polarimetric PHASR Scanner by comparing calibration results obtained from multiple angle sets with an 18-angle reference. Using only three optimized angles, the relative errors in the calibrated incident field and the system Jones matrix remained below 10% relative to the reference calibration, substantially reducing the acquisition requirements for a calibration set. We also present a framework for extracting the Jones matrix of a sample from measurements acquired at two independent sample rotation angles. This approach is validated using a rotating x-cut lithium niobate crystal, where the improved leaky models mitigate the systematic error observed under an idealpolarizer assumption. Finally, we used the calibrated PHASR Scanner to map birefringent samples by eigendecomposition of the measured Jones matrices. Although the principal axes of the samples are unknown *a priori*, measurements at two sample orientations enable recovery of the intrinsic reflection coefficients along the two optical axes.

## II. Polarimetric THz Scanner

**Fig. 1.**
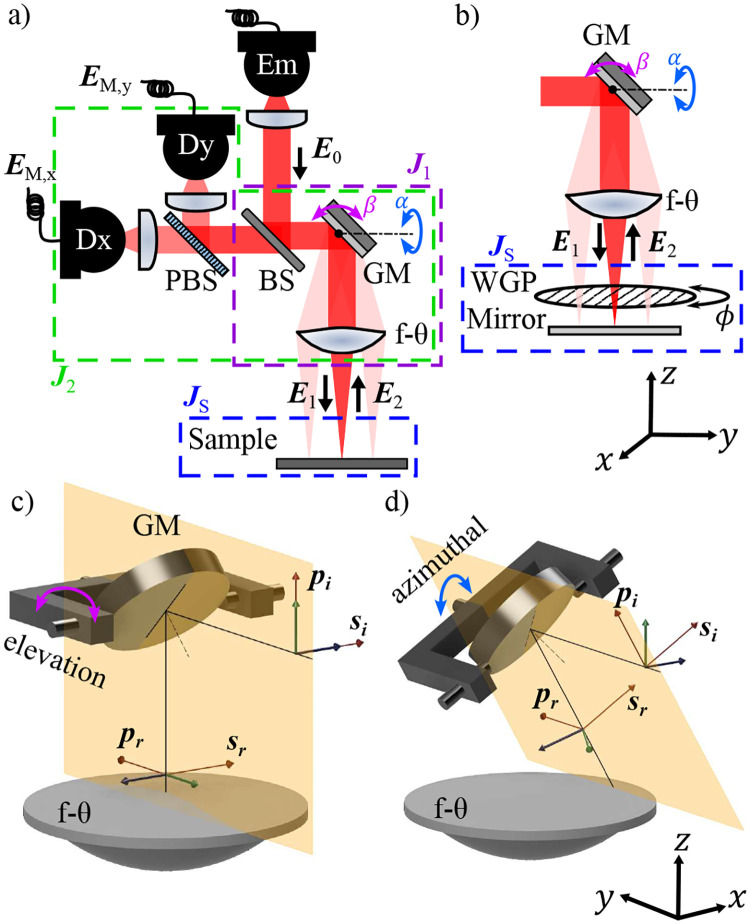
(a) Schematic of the polarimetric THz PHASR Scanner. Em: emitter, Dx and Dy: x- and y-polarized detectors, GM: gimbal mirror, BS: high-resistivity silicon beam splitter, PBS: polarizing beam splitter, f-θ: custom-made f-θ lens, $E_{0}$: emitted electric field, $E_{1}$: incident electric field on the sample, $E_{2}$: reflected electric field from the sample, $E_{\text{M}}$: measured electric field by the two orthogonal channels, $J_{1}$: Jones matrix of the scanner on the emitter path, $J_{2}$: Jones matrix of the scanner on detection path, $J_{\text{S}}$: Jones matrix of the sample, α and β are the azimuthal and elevation rotation angle of the GMm, respectively. (b) In-situ full calibration of the scanner using a rotating WGP placed in front of a mirror at the sample position. (c) and (d) show the polarization states of the incident and reflected THz beam, when the GM mirror was not deflected (c) and with deflection in the azimuthal direction (d). The plane of incidence is also shown. The blue and green arrows indicate the basis for the polarization state reflected from the silicon BS - $s_{i}$ and $p_{i}$ - and at location of the f-θ lens - $s_{r}$ and $p_{r}$ -, while the red arrows show the state of the polarization relative to the plane of incidence.

The schematic diagram of the optical components of the polarimetric THz PHASR Scanner is shown in Fig. 1(a). The scanner consists of a fiber-coupled photoconductive antenna (PCA) emitter Em rotated to a 45° angle and a pair of orthogonally oriented PCA detectors, Dx and Dy, to record the two perpendicular bases of the polarization state of the electric field. The detector PCAs sample the time-domain waveform using the Electronically Controlled OPtical Sampling (ECOPS) technique [56] (TeraFlash smart, Toptica Photonics AG, Germany). The collimated THz electric field $E_{0}$, generated by Em, is reflected from a high-resistivity silicon beam splitter (BS), and is steered across a custom-made telecentric f-θ lens via a motorized gimbal mirror (GM). As shown in Figs. 1(c) and (d), the rotation of the motors vary the incident angle of the beam on the GM. Consequently, the s- and p-polarization components of the reflected pulse from the GM change with respect to the fixed coordinate system. The azimuthal rotation α, which steers the beam along the x-axis of the FOV affects both s- and p-polarizations of the beam, while the elevation rotation β, which steers the beam along the y-axis, affects only the p-polarization [35]. The f-θ lens focuses the steered beam on the sample, ensuring a constant time-of-arrival (ToA) and spot size over the FOV. The Jones matrix description of the beam path from the emitter to the sample is

(1) $$\left[\begin{array}{l} E_{1,x}\\ E_{1,y} \end{array}\right]=J_{1}\left[\begin{array}{l} E_{0,x}\\ E_{0,y} \end{array}\right],$$

where $J_{1}$ is the Jones matrix of the scanner on the emittersample path, and $E_{1}$ is the incident electric field on the sample. The reflected electric field of the sample $E_{2}$ passes the f-θ lens and GM path, and after transmitting through the BS, is divided into two orthogonal polarization bases by a polarizing beam splitter (PBS). The PBS is a WGP, whose optical axis has a 45° angle with respect to the beam path and divides the beam into its orthogonal components to be recorded by Dx and Dy. The recorded electric field $E_{\text{M}}$ is described by

(2) $$E_{\text{M}}=\left[\begin{array}{l} E_{\text{M},x}\\ E_{\text{M},y} \end{array}\right]=J_{2}J_{\text{S}}\left[\begin{array}{l} E_{1,x}\\ E_{1,y} \end{array}\right],$$

where $J_{\text{S}}$ is the Jones matrix of the sample and $J_{2}$ is the Jones matrix of the scanner on the detection path, including the changes in polarization due to beam steering, the response of the optical components, and the transimpedance amplifiers in Dx and Dy, and is defined generally by

(3) $$J_{2}=\left[\begin{array}{ll} s_{xx} & s_{xy}\\ s_{yx} & s_{yy} \end{array}\right].$$

For a given measured THz field $E_{\text{M}}$, $J_{2}$ and $E_{1}$ are needed to extract $J_{\text{S}}$. Note that extracting $E_{0}$ and $J_{1}$ is not required for sample characterization, and $J_{1}E_{0}$ can be replaced by $E_{1}$. In the following section, the calibration framework of $J_{2}$ and $E_{1}$ is described.

## III. Calibration

### A. Calibrating polarizer

**Fig. 2.**
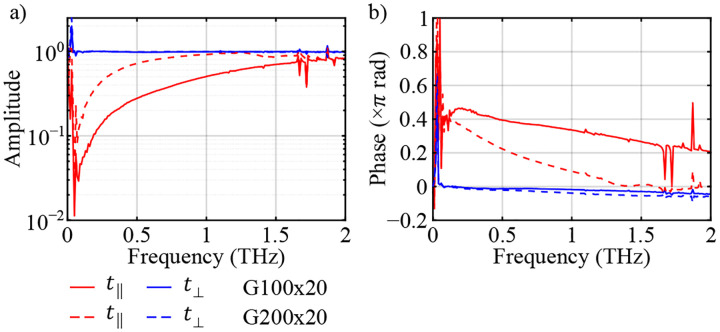
(a) Amplitude and (b) phase of the transmission component of the parallel (red) and perpendicular (blue) polarization direction with respect to the orientation of the WGP is shown for G100×20 (solid lined) and G200×20 (dashed lines) polarizers.

Fig. 1(b) schematically shows the *in situ* calibration method by placing a rotating WGP in front of a mirror, as a known reference. A simple and idealized Jones matrix description of the WGP is given as

(4) $$J_{\text{P}}=\left[\begin{array}{ll} 1 & 0\\ 0 & 0 \end{array}\right],$$

where the polarizer fully transmits the electric field perpendicular to the orientation of its wires and blocks the parallel polarization. In other words, the loss and leakage of the polarizer is ignored. However, wire-grid polarizers are imperfect and have frequency-dependent transmission coefficients,

(5) $$J_{\text{P}}=\left[\begin{array}{cc} t_{⊥} & 0\\ 0 & t_{\|} \end{array}\right],$$

where $t_{⊥}$ and $t_{\|}$ are the complex transmission coefficients of the polarizations perpendicular and parallel to the orientation of the wires. The frequency-dependent response of free-standing WGPs depends on the conductivity, diameter d and period p of the wires, and the operating wavelength λ. Given d≪λ, and the high conductivity of the metallic wires, the loss of the polarizer can be neglected. However, commercially available THz WGPs have p in the same order of magnitude as λ, which gives rise to a non-negligible leakage of the parallel polarization to the orientation of the wires [53]. With the assumption of a lossless and leaky polarizer, (5) can be substituted by [57]

(6a) $$J_{\text{P}}=\left[\begin{array}{cc} 1 & 0\\ 0 & \eta \;\text{exp}(\text{j}\delta ) \end{array}\right],$$

(6b) $$\eta =\left|t_{\|}\right|,$$

(6c) $$\delta =∠t_{\|},$$

where η and δ are the frequency-dependent extinction coefficient and phase retardance of the WGP. In this work, two free-standing tungsten WGP were used as the calibrating polarizer, with a wire diameter of 20μm and a wire spacing of 100μm and 200μm, namely G100×20 and G200×20, respectively (Microtech Instrument Inc.). Only one polarizer at a time is used for the calibration, and the use of two polarizers is for comparison purposes. Transmission measurements were performed using a pair of fiber-coupled PCAs (TeraSmart, Menlo Systems Inc. Newton, NJ, USA) to extract $t_{\|}$ and $t_{⊥}$. Since PCAs produce quasi-linearly polarized light [58], a secondary G100×20 WGP was placed on the beam path with its wires perpendicular to the main polarization axis of the PCAs to minimize polarization cross-talks [59]. A reference pulse ($E_{\text{ref}}$) in the absence of the sample WGP, and transmission measurements of the sample WGP at rotation angles 0° $\left(E_{\|}\right)$ and 90° $\left(E_{⊥}\right)$ were recorded. The complex transmission coefficients $t_{⊥}$ and $t_{\|}$ were extracted by dividing $E_{\|}$ and $E_{⊥}$, respectively, by $E_{\text{ref}}$, as shown in Fig. 2. As can be seen, $t_{⊥}$ of both polarizers is nearly 1 and, therefore, the loss is ignored. However, the leakage of both polarizers is highly sensitive to frequency, reaching nearly 1 (100%) at 1 THz and 1.8 THz in G200×20 and G100×20, respectively. Since THz-TDS systems are inherently broadband, using the frequency-dependent leakage of the calibrating polarizer is essential for an accurate calibration of the system. The extracted η and δ were used in the calibration equations, discussed next in Section III-B. As an alternative to the characterization of the calibrating polarizer by separate transmission measurements, η and δ can be obtained together with the remaining calibration parameters via optimization techniques [60] or impedance models [53].

### B. Calibration Equations

**Fig. 3.**
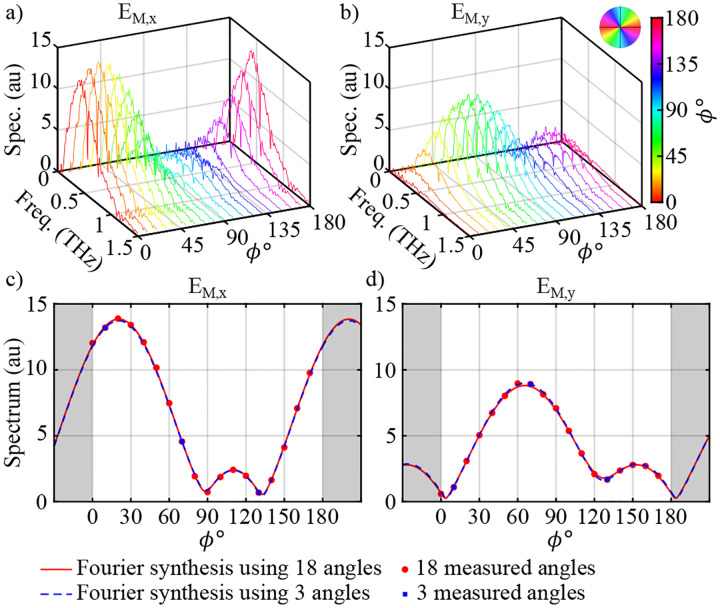
Recorded spectra of (a) x- and (b) y-polarized channels as a function of rotation angle of the WGP. The spectra are spatially averaged over a 9 mm vertical line in the center of the FOV. Recorded spectra and the Fourier synthesis curves using 18 measurements with $\Delta \phi =10^{\circ }$ (red solid line) and 3 measurements with $\Delta \phi =60^{\circ }$ (blue dashed line) measured by (c) x- and (d) y- polarized channels at f=0.5 THz.

In this section, we describe an analytical relationship for the calibration parameters of the scanner. It can be shown that the relative error of the measured electric field $E_{\text{M}}$ in the case of having a mirror in the sample position is proportional to $\eta^{2}$ (see Section I of the Supplementary Material). As shown in Fig. 1(b), the calibrating WGP was placed in front of a gold coated mirror in the sample position. Two-channel THz-TDS measurements were recorded while the WGP was rotated at every 10° between 0° and 170°. The rotation angle of the calibrating WGP, ϕ, is defined such that at $\phi =0^{\circ }$ the wires are along the y-axis, and ϕ increases with counterclockwise rotation of the WGP. Figs. 3(a) and (b) show the spectra of the measured data $E_{\text{M},\text{x}}$ and $E_{\text{M},\text{y}}$ in the center of the field of view (FOV) as a function of the rotation angle ϕ.

By substituting (6a) into $J_{\text{S}}$ in (2) and considering the rotation of the WGP by $\phi_{0}$, the description of the calibration measurement can be given as

(7a) $$E_{\text{M}}\left(\phi_{0}\right)=J_{2}R\left(-\phi_{0}\right)J_{\text{P}}J_{\text{M}}J_{\text{P}}R\left(\phi_{0}\right)E_{1},$$

(7b) $$R\left(\phi_{0}\right)=\left[\begin{array}{cc} \text{cos}\;\phi_{0} & \text{sin}\;\phi_{0}\\ -\text{sin}\;\phi_{0} & \text{cos}\;\phi_{0} \end{array}\right],$$

(7c) $$\begin{array}{c} J_{\text{M}}=\left[\begin{array}{cc} -1 & 0\\ 0 & -1 \end{array}\right], \end{array}$$

where R(ϕ) is the rotation matrix and $J_{\text{M}}$ is the Jones matrix of the mirror in the fixed coordinate system [61]–[63]. The objective of the calibration is to extract $J_{2}$ and $E_{1}$ (i.e., 6 total unknowns). Because the gain of the x and y channel PCA detectors can be different, there is a multiplicative nonuniqueness between $J_{2}$ and $E_{1}$. To remove this ambiguity, $s_{xx}$ in (3) is factored out of $J_{2}$ and normalized in $E_{1}$, thereby fixing the overall scale without loss of generality. Therefore, the new calibration equation is defined as

(8a) $$E_{\text{M}}\left(\phi_{0}\right)=J_{2}^{\prime }R\left(-\phi_{0}\right)J_{\text{P}}J_{\text{M}}J_{\text{P}}R\left(\phi_{0}\right)E_{1}^{\prime },$$

(8b) $$J_{2}^{\prime }=\frac{1}{s_{xx}}J_{2}=\left[\begin{array}{cc} 1 & s_{1}\\ s_{2} & s_{3} \end{array}\right],$$

(8c) $$\begin{array}{c} E_{1}^{\prime }=s_{xx}E_{1}=\left[\begin{array}{l} E_{1,x}^{\prime }\\ E_{1,y}^{\prime } \end{array}\right] \end{array}.$$

This factorization reduces the number of calibration parameters to 5, while it does not affect $E_{\text{M}}$ and $J_{\text{S}}$. In (8a), we define the calibration sample’s Jones matrix, $J_{\text{S}}$, by,

(9) $$J_{\text{S}}=R\left(-\phi_{0}\right)J_{\text{P}}J_{\text{M}}J_{\text{P}}R\left(\phi_{0}\right),$$

where the columns of $J_{\text{S}}$ are

(10a) $$J_{{\text{S}}_{:,1}}=-\left[\begin{array}{c} {\text{cos}}^{2}\left(\phi_{0}\right)+\zeta {\text{sin}}^{2}\;\left(\phi_{0}\right)\\ (1-\zeta )\text{cos}\left(\phi_{0}\right)\text{sin}\left(\phi_{0}\right) \end{array}\right],$$

(10b) $$\begin{array}{cc} & J_{{\text{S}}_{:,2}}=-\left[\begin{array}{c} (1-\zeta )\text{cos}\left(\phi_{0}\right)\text{sin}\left(\phi_{0}\right)\\ \zeta {\;\text{cos}}^{2}\left(\phi_{0}\right)+{\text{sin}}^{2}\left(\phi_{0}\right) \end{array}\right], \end{array}$$

and ζ is the square of $J_{{\text{P}}_{22}}$ element in (6a), defined by

(11) $$\zeta =\eta^{2}\;\text{exp}(2\text{j}\delta ).$$

The calibration measurements as a function of the rotation angle of the WGP are therefore given by

(12a) $$E_{\text{M},x}\left(\phi_{0}\right)=-\left(\left(E_{1,x}^{\prime }+\zeta s_{1}E_{1,y}^{\prime }\right){\text{cos}}^{2}\left(\phi_{0}\right)\right.\;+\left(s_{1}E_{1,x}^{\prime }+E_{1,y}^{\prime }\right)(1-\zeta )\text{cos}\left(\phi_{0}\right)\text{sin}\left(\phi_{0}\right)\;\left.+\left(\zeta E_{1,x}^{\prime }+s_{1}E_{1,y}^{\prime }\right){\text{sin}}^{2}\left(\phi_{0}\right)\right)$$

(12b) $$E_{\text{M},y}\left(\phi_{0}\right)=-\left(s_{2}E_{1,x}^{\prime }+\zeta s_{3}E_{1,y}^{\prime }\right){\text{cos}}^{2}\left(\phi_{0}\right)\;-\left(s_{3}E_{1,x}^{\prime }+s_{2}E_{1,y}^{\prime }\right)(1-\zeta )\text{cos}\left(\phi_{0}\right)\text{sin}\left(\phi_{0}\right)\;\left.-\left(\zeta s_{2}E_{1,x}^{\prime }+s_{3}E_{1,y}^{\prime }\right){\text{sin}}^{2}\left(\phi_{0}\right)\right).$$

Next, we derive the following system of equations by separating the known angle-dependent coefficients from the unknown calibration parameters in (12a) and (12b),

(13a) $$E_{\text{M}}\left(\phi_{0}\right)=A_{2\times 3}P{\left(\phi_{0}\right)}_{3\times 1},$$

(13b) $$P{\left(\phi_{0}\right)}_{3\times 1}=-\left[\begin{array}{c} {\text{cos}}^{2}\;\phi_{0}\\ \text{cos}\;\phi_{0}\;\text{sin}\;\phi_{0}\\ {\text{sin}}^{2}\;\phi_{0} \end{array}\right],$$

(13c) $$A_{1,:}={\left[\begin{array}{c} E_{1,x}^{\prime }+\zeta s_{1}E_{1,y}^{\prime }\\ \left(s_{1}E_{1,x}^{\prime }+E_{1,y}^{\prime }\right)(1-\zeta )\\ \zeta E_{1,x}^{\prime }+s_{1}E_{1,y}^{\prime } \end{array}\right]}^{T},$$

(13d) $$\begin{array}{c} A_{2,:}={\left[\begin{array}{c} s_{2}E_{1,x}^{\prime }+\zeta s_{3}E_{1,y}^{\prime }\\ \left(s_{3}E_{1,x}^{\prime }+s_{2}E_{1,y}^{\prime }\right)(1-\zeta )\\ \zeta s_{2}E_{1,x}^{\prime }+s_{3}E_{1,y}^{\prime } \end{array}\right]}^{T}, \end{array}$$

where $P\left(\phi_{0}\right)$ is the vector of angular coefficients and A is the auxiliary matrix containing equations that give the 5 calibration parameters. Therefore, to estimate these calibration parameters, at least 3 separate measurements, $E_{\text{M}}$ should be captured. Inverting $P\left(\phi_{0}\right)$ and multiplying by $E_{\text{M}}$ at these three measurement angles directly calculates the members of $A_{2\times 3}$.

### C. Alternative Form of Calibration Equations

Alternatively, the angular coefficients can be reformulated using the following trigonometric identities:

(14a) $${\text{sin}}^{2}\;\phi =\frac{1-\text{cos}\;2\phi }{2},$$

(14b) $${\text{cos}}^{2}\;\phi =\frac{1+\text{cos}\;2\phi }{2},$$

(14c) $$\text{sin}\;\phi \;\text{cos}\;\phi =\frac{\text{sin}\;2\phi }{2}.$$

Using these relationships, (10) can be reformulated as

(15a) $$J_{{\text{S}}_{:,1}}=-\frac{1}{2}\left[\begin{array}{c} 1+\zeta +\left(1-\zeta \right)\text{cos}\left(2\phi_{0}\right)\\ \left(1-\zeta \right)\text{sin}\left(2\phi_{0}\right) \end{array}\right],$$

(15b) $$\begin{array}{c} J_{{\text{S}}_{:,2}}=-\frac{1}{2}\left[\begin{array}{c} (1-\zeta )\text{sin}\left(2\phi_{0}\right)\\ 1+\zeta -(1-\zeta )\text{cos}\left(2\phi_{0}\right) \end{array}\right]. \end{array}$$

And the calibration measurements in (12) can be rewritten such that

(16a) $$E_{\text{M},x}\left(\phi_{0}\right)=-\frac{1}{2}\left(\left(E_{1,x}^{\prime }+s_{1}E_{1,y}^{\prime }\right)(1+\zeta )\right.\;+\left(E_{1,x}^{\prime }-s_{1}E_{1,y}^{\prime }\right)(1-\zeta )\text{cos}\left(2\phi_{0}\right)\;\left.+\left(s_{1}E_{1,x}^{\prime }+E_{1,y}^{\prime }\right)(1-\zeta )\text{sin}\left(2\phi_{0}\right)\right),$$

(16b) $$E_{\text{M},y}\left(\phi_{0}\right)=-\frac{1}{2}\left(\left(s_{2}E_{1,x}^{\prime }+s_{3}E_{1,y}^{\prime }\right)(1+\zeta )\right.\;+\left(s_{2}E_{1,x}^{\prime }-s_{3}E_{1,y}^{\prime }\right)(1-\zeta )\text{cos}\left(2\phi_{0}\right)\;\left.+\left(s_{3}E_{1,x}^{\prime }+s_{2}E_{1,y}^{\prime }\right)(1-\zeta )\text{sin}\left(2\phi_{0}\right)\right).$$

Equivalent to (13), the angle-dependent coefficients can be separated to form a new system of equations, given by,

(17a) $$E_{\text{M}}\left(\phi_{0}\right)=B_{2\times 3}Q{\left(\phi_{0}\right)}_{3\times 1},$$

(17b) $$Q_{3\times 1}\left(\phi_{0}\right)=-\frac{1}{2}\left[\begin{array}{c} 1\\ \text{cos}\;2\phi_{0}\\ \text{sin}\;2\phi_{0} \end{array}\right],$$

(17c) $$B_{1,:}={\left[\begin{array}{c} \left(E_{1,x}^{\prime }+s_{1}E_{1,y}^{\prime }\right)(1+\zeta )\\ \left(E_{1,x}^{\prime }-s_{1}E_{1,y}^{\prime }\right)(1-\zeta )\\ \left(s_{1}E_{1,x}^{\prime }+E_{1,y}^{\prime }\right)(1-\zeta ) \end{array}\right]}^{T},$$

(17d) $$\begin{array}{c} B_{2,:}={\left[\begin{array}{c} \left(s_{2}E_{1,x}^{\prime }+s_{3}E_{1,y}^{\prime }\right)(1+\zeta )\\ \left(s_{2}E_{1,x}^{\prime }-s_{3}E_{1,y}^{\prime }\right)(1-\zeta )\\ \left(s_{3}E_{1,x}^{\prime }+s_{2}E_{1,y}^{\prime }\right)(1-\zeta ) \end{array}\right]}^{T}. \end{array}$$

Note that rank(P)=1 and rank(Q)=1, and therefore, the solutions to $A_{2\times 3}$ and $B_{2\times 3}$ are not unique (i.e., (13) and (17) are underdetermined systems of equations). Therefore, to satisfy the minimum rank criterion, N(N≥3) measured $E_{\text{M}}\left(\phi_{n}\right)$ with different and independent angles ($\phi_{n}\neq \phi_{m}+k\pi ,\forall m,n,k\in N$), are concatenated column-wise and (17a) and (13a) are modified to

(18a) $$E_{\text{M}}(\phi {)}_{2\times N}=A_{2\times 3}{\left[P\left(\phi_{1}\right),\cdots ,P\left(\phi_{N}\right)\right]}_{3\times N},$$

(18b) $$E_{\text{M}}(\phi {)}_{2\times N}=B_{2\times 3}{\left[Q\left(\phi_{1}\right),\cdots ,Q\left(\phi_{N}\right)\right]}_{3\times N},$$

(18c) $$E_{\text{M}}(\phi {)}_{2\times N}={\left[E_{\text{M}}\left(\phi_{1}\right),\cdots ,E_{\text{M}}\left(\phi_{N}\right)\right]}_{2\times N},$$

where ϕ refers to a series of angle measurements, while $\phi_{n}$ refers to a specific polarizer rotation angle. Since Q contains the orthogonal bases of P, (13) and (17) can be linked by a transformation matrix $T_{3\times 3}$ defined as

(19a) $$T_{3\times 3}Q_{3\times N}\left(\phi \right)=P_{3\times N}\left(\phi \right),$$

(19b) $$A_{2\times 3}T_{3\times 3}=B_{2\times 3},$$

(19c) $$\begin{array}{c} T_{3\times 3}=\frac{1}{2}\left[\begin{array}{ccc} 1 & 1 & 0\\ 0 & 0 & 2\\ 1 & -1 & 0 \end{array}\right] \end{array}.$$

The two forms of the auxiliary matrices A and B can be calculated by right-multiplication of the pseudoinverse of the matrix of angular coefficients to both sides of (18a) and (18b)

(20a) $$A_{2\times 3}=E_{\text{M}}(\phi {)}_{2\times N}P(\phi {)}^{†},$$

(20b) $$B_{2\times 3}=E_{\text{M}}(\phi {)}_{2\times N}Q(\phi {)}^{†},$$

where † indicates the Moore-Penrose pseudo inverse operation. Since harmonic-basis terms appear in (17b), (20b) can also be solved by Fourier series analysis given by

(21a) $$B_{i1}=\frac{1}{N}\sum_{i=1}^{N}-2E_{\text{M}}\left(\phi_{i}\right),$$

(21b) $$B_{i2}=\frac{2}{N}\sum_{i=1}^{N}-2E_{\text{M}}\left(\phi_{i}\right)\text{cos}\left(2\phi_{i}\right),$$

(21c) $$B_{i3}=\frac{2}{N}\sum_{i=1}^{N}-2E_{\text{M}}\left(\phi_{i}\right)\text{sin}\left(2\phi_{i}\right).$$

Although (20b) and (21) are numerically equivalent for a given series of equally spaced angles, the latter provides a more intuitive solution based on the Fourier sum of the calibration angles used. In addition, the Fourier analysis solution imposes equally spaced sampling of the rotation angles over a period of π, which is shown to be the optimized choice of angles in Sub-section III-E. Figs. 3(c) and (d) show the measured $E_{\text{M},x}$ and $E_{\text{M},y}$, respectively, as a function of the rotation angle of the calibrating WGP at f=0.5 THz for angular steps of $\Delta \phi =10^{\circ }$ (red circles). Using the Fourier series interpretation in (21), we can synthesize the B coefficients for a given N≥3 choice of angles with the angle spacing of $\frac{\pi }{N}$. The Fourier synthesized line extracted from the 18 angles is shown as red solid line in Figs. 3(c) and (d). Additionally, the Fourier synthesized line (blue dashed curve) is shown for coefficients extracted from three angles with $\Delta \phi =60^{\circ }$ (blue squares). The Fourier synthesized lines extracted using 3 angles closely match using 18 angles.

### D. Calibration Parameters

The calibration parameters, i.e., $E_{1,x}^{\prime },E_{1,y}^{\prime },s_{1},s_{2}$ and $s_{3}$, are non-linearly related to the elements of A and B. However, we can derive a semi-analytical solution to calculate the calibration parameters directly. Because the two matrices A and B are linearly related by the transformation T in (19), we only present the solution of the calibration parameters using B in this section. First, B is decomposed by multiplication of a calibration parameter matrix, $B_{0}$, and a matrix containing the leakage-dependent terms, Z, such that

(22a) $$B=B_{0}Z,$$

and

(22b) $$Z=\left[\begin{array}{ccc} 1+\zeta & 0 & 0\\ 0 & 1-\zeta & 0\\ 0 & 0 & 1-\zeta \end{array}\right],$$

(22c) $$\begin{array}{c} B_{0}={\left[\begin{array}{ll} E_{1,x}^{\prime }+s_{1}E_{1,y}^{\prime } & s_{2}E_{1,x}^{\prime }+s_{3}E_{1,y}^{\prime }\\ E_{1,x}^{\prime }-s_{1}E_{1,y}^{\prime } & s_{2}E_{1,x}^{\prime }-s_{3}E_{1,y}^{\prime }\\ s_{1}E_{1,x}^{\prime }+E_{1,y}^{\prime } & s_{3}E_{1,x}^{\prime }+s_{2}E_{1,y}^{\prime } \end{array}\right]}^{T}, \end{array}$$

where $B_{0}$ is independent of the calibrating polarizer and can be calculated from $B_{0}=BZ^{-1}$. The analytical solution for the calibration parameters can be derived using five of the six elements of $B_{0}$ (see Section II of the Supplementary Materials) using the following relationships

(23a) $$E_{1,x}^{\prime }=\frac{B_{0_{11}}+B_{0_{12}}}{2},$$

(23b) $$E_{1,y}^{\prime }=\frac{B_{0_{11}}^{2}-B_{0_{12}}^{2}}{2\left(B_{0_{13}}+\sqrt{\Delta }\right)},$$

(23c) $$s_{1}=\frac{B_{0_{13}}+\sqrt{\Delta }}{B_{0_{11}}+B_{0_{12}}},$$

(23d) $$s_{2}=\frac{B_{0_{21}}+B_{0_{22}}}{B_{0_{11}}+B_{0_{12}}}$$

(23e) $$s_{3}=\frac{\left(B_{0_{21}}+B_{0_{22}}\right)\left(B_{0_{13}}+\sqrt{\Delta }\right)}{B_{0_{11}}^{2}-B_{0_{12}}^{2}},$$

where Δ is the discriminant of a quadratic equation, which is given by

(24) $$\Delta =B_{0_{13}}^{2}+B_{0_{12}}^{2}-B_{0_{11}}^{2}.$$

Due to the inherent ambiguity of square root, $\sqrt{\Delta },E_{1,y}^{\prime },s_{1}$, and $s_{3}$ have two sets of solutions. The choice of the correct root can be constrained by the unused element $B_{0_{23}}$, i.e., by substituting the analytical equations (23) into $B_{023}$, the two possible numeric values are compared with $B_{0_{23}}$ (see Section III of the Supplementary Material).

### E. Choice of Polarizer Rotation Angles

Since the aim is to minimize the number of measurements needed for the calibration of the instrument, it is important to ensure that the system of equations in (18) remains well-conditioned. In practice, this can be achieved by choosing the rotation angles ϕ such that the matrices $P(\phi {)}_{3\times N}$ and $Q(\phi {)}_{3\times N}$ have a minimized condition number, thereby reducing the sensitivity of measurement to errors or uncertainty perturbations. The condition numbers can be given by

(25a) $$\kappa_{P}(\phi )=\|P\left(\phi \right){\|}_{2}{\left\|P^{†}\left(\phi \right)\right\|}_{2},$$

(25b) $$\begin{array}{cc} & \kappa_{Q}(\phi )=\|Q(\phi ){\|}_{2}{\left\|Q^{†}(\phi )\right\|}_{2}. \end{array}$$

For N=3 angles, the minimized $\kappa_{P}(\phi )$ and $\kappa_{Q}(\phi )$ are achieved by different sets of angles. Here, we find that $\kappa_{P}(\phi )$ is minimized by non-equally spaced angles $\phi =\left[23^{\circ },67^{\circ },135^{\circ }\right]$ and $\phi =\left[157^{\circ },113^{\circ },45^{\circ }\right]$, while $\kappa_{Q}(\phi )$ is minimized by any sets of equidistant angles with $\Delta \phi =60^{\circ }$ over period of π, which was also imposed by the Fourier analysis method. Intuitively, Q contains orthogonal bases and should be a better choice compared to P.

**Fig. 4.**
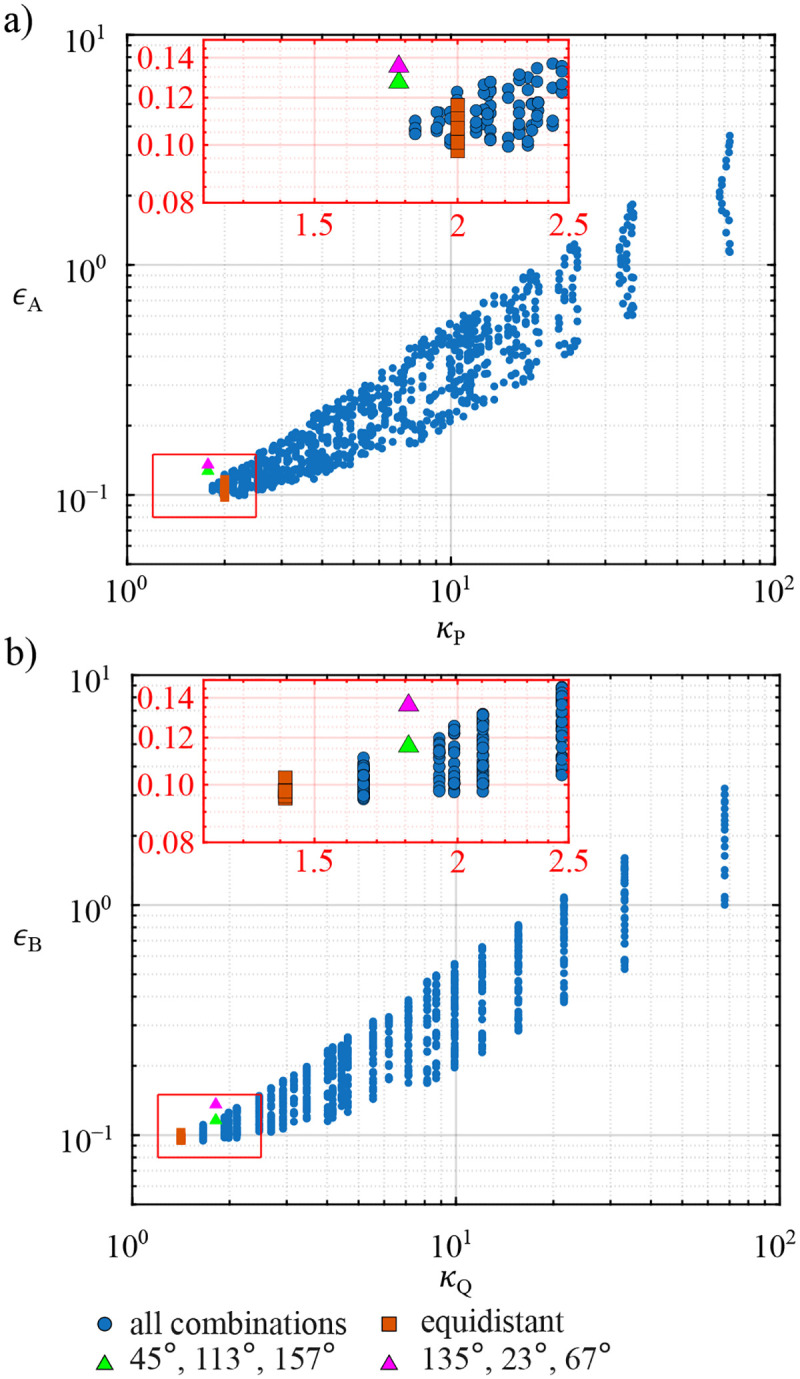
a) The relative error of A using 3 angles as a function of the condition number of P. b) The relative error of B using 3 angles as a function of the condition number of Q. The equidistant angles over a period of π, minimizing the $\kappa_{Q}$ are shown as red rectangles, and the two sets of angles minimizing $\kappa_{P}$ are shown as green and magenta triangles.

To compare the two alternative calibration formulations, 18 measurements with $\Delta \phi =10^{\circ }$ were recorded. In addition, the two sets of angles that result in the minimized $\kappa_{P}(\phi )$ were measured too. The matrices B and A are calculated from (20) using the 18 angles as the ground truth (shown with the hat sign). The combinations of choosing 3 from the 18 angles and, in addition, the two sets [23°, 67°, 135°] and [157°, 113°, 45°] were used to calculate A and B. The relative errors of the two auxiliary matrices obtained by various combinations of 3 angles with respect to their ground truths, $\overset{ˆ}{A}$ and $\overset{ˆ}{B}$, are calculated by

(26a) $$\epsilon_{A}=\frac{{\left\|\langle |A-\overset{ˆ}{A}|\rangle_{x,y,f}\right\|}_{2}}{{\left\|\langle |\overset{ˆ}{A}|\rangle_{x,y,f}\right\|}_{2}},$$

and

(26b) $$\epsilon_{B}=\frac{{\left\|\langle |B-\overset{ˆ}{B}|\rangle_{x,y,f}\right\|}_{2}}{{\left\|\langle |\overset{ˆ}{B}|\rangle_{x,y,f}\right\|}_{2}},$$

where $\langle .\rangle_{x,y,f}$ indicates the average along the frequency and spatial dimensions. Figs. 4(a) and (b) show the relative error of A and B calculated using three angles as a function of the condition number of their corresponding angular matrix P and Q, respectively. It is evident that the relative errors increase with the condition numbers of the angular matrices. Additionally, equidistant angle sets, which result in the minimized $\kappa_{Q}$, lead to the minimum relative error in either of the methods. However, the two sets of angles, resulting in the minimized $\kappa_{P}$, lead to slightly larger relative errors. This indicates the advantage of choosing the angular matrix Q, which has orthogonal basis elements. It should be mentioned that minimizing the condition number does not necessarily lead to the most optimum choice of angles, as the signal-to-noise ratio (SNR) of the measurements is a function of ϕ. However, estimating the relationship between the relative error of $E_{\text{M}}$ and ϕ requires prior knowledge of A or B. Therefore, for an uncharacterized system, this method ensures a well-conditioned system of equations by minimizing the sensitivity to propagation of error. This approach provides a practical guide to ensure that the resulting set of calibration angles is among the best possible choices.

**Fig. 5.**
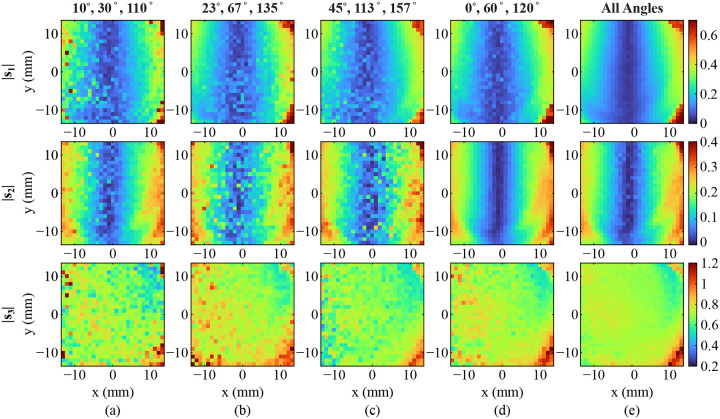
The spatial distribution of the elements of $J_{2}^{\prime }$ calibrated using (a) a random set of angles 10°, 30°, 110°, (b) 23°, 67°, 135° and (c) 45°, 113°, 157° that minimize $\kappa_{P}$, (d) an equidistant angle set minimizing $\kappa_{Q}$, (e) and the ground truth using all 18 angles. The pseudo-color shows the average of the amplitude of the parameters between 0.4 and 0.5 THz.

To further investigate the effect of the choice of polarizer rotation angles on the calibrated Jones matrix of the system $J_{2}^{\prime }$ and the incident electric field $E_{1}^{\prime }$, calibration was performed using 4 subsets of angles and the reference (ground truth) using 18 angles. Fig. 5 compares the three calibration parameters $s_{1}$, $s_{2}$, and $s_{3}$ calculated from one random set of angles, shown in Fig. 5(a), the two sets of angles minimizing $\kappa_{P}$ in Figs. 5(b) and Figs. 5(c), one set of equidistant angle that minimizes $\kappa_{Q}$ in Figs. 5(d), and the ground truth using all 18 angles in Figs. 5(e). The spatial distributions of $s_{1}$, $s_{2}$, and $s_{3}$ are the average of the amplitude between 0.4 and 0.5 THz. It is evident that the equidistant set is the most similar to the ground truth compared to the other sets. Here $s_{1}$ and $s_{2}$ represent the cross-talk of the two channels normalized by the gain of the x-channel, as defined in (8b). As the beam is steered by the GM, the polarization is rotated within the return path, increasing the cross-talk and creating a symmetric shape in $\left|s_{1}\right|$ and $\left|s_{2}\right|$. Moreover, $s_{3}$ is the ratio of the gain of the y-channel to the x-channel, which shows a relatively uniform amplitude over space and has only a slight variation between the left and right sides of the FOV.

**Fig. 6.**
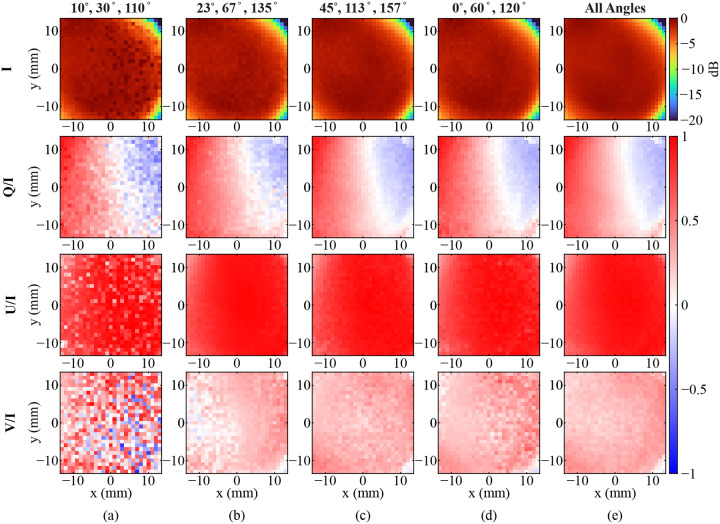
The spatial distribution of the Stokes vector elements of $E_{1}^{\prime }$ calibrated using (a) a random set of angles 10°, 30°, 110°, (b) 23°, 67°, 135° and (c) 45°, 113°, 157° that minimize $\kappa_{P}$, (d) an equidistant angle set, (e) and the ground truth using all 18 angles. The pseudo-colors show the average of the amplitude of the parameters between 0.4 and 0.5 THz.

The Stokes vector representation of the incident electric field $E_{1}^{\prime }$ is given as

(27) $$\left[\begin{array}{c} I\\ Q\\ U\\ V \end{array}\right]=\left[\begin{array}{c} {\left|E_{1,x}^{\prime }\right|}^{2}+{\left|E_{1,y}^{\prime }\right|}^{2}\\ {\left|E_{1,x}^{\prime }\right|}^{2}-{\left|E_{1,y}^{\prime }\right|}^{2}\\ 2R\left\{E_{1,x}^{\prime }E_{1,y}^{\prime *}\right\}\\ -2I\left\{E_{1,x}^{\prime }E_{1,y}^{\prime *}\right\} \end{array}\right],$$

where I is the total intensity of the electric field, Q is the difference between the horizontal and vertical, U is the difference between 45° and −45°, and V is the difference between right-handed and left-handed circular polarizations. As a common practice, Q, U and V can be normalized by I to limit their values between −1 and 1. The Stokes vector of $E_{1}^{\prime }$ is shown in Fig. 6(a) calibrated using a random set of 3 angles, using the two sets that minimize $\kappa_{P}$ in Figs. 6(b) and (c), using an equidistant angle set in Fig. 6(d), and using all 18 angles as the ground truth in Fig. 6(e). Both the equidistant angle set and the [45°, 113°, 157°] set match closely the ground truth. Since the rotation angle of the polarizer in 135° has parallel wires with respect to the polarization of the emitter, the angle set [23°, 67°, 135°] has a lower SNR compared to that of [45°, 113°, 157°], which is evident in Figs. 6(b) and (c).

The calibration of the incident electric field $E_{1}^{\prime }$ shows a uniform intensity I throughout the FOV, and only the corner pixels have a reduced I. As discussed in Section II, the polarization state of the incident electric field varies with azimuthal and elevation movements of the GM. This change in polarization is evident in the spatial distribution of Q in Fig. 6. Since the emitter is rotated to a 45° position, and the rotation of the GM is limited, the 45° polarization is dominant compared to the −45°, showing a saturated value of U throughout the FOV. Similarly, the handedness of the incident polarization shows a relatively uniform value over the FOV.

We define the relative error of $J_{2}^{\prime }$ and $E_{1}^{\prime }$ when calibrated using various angle sets by

(28a) $$\epsilon_{E_{1}^{\prime }}=\frac{{\left\|\langle |E_{1}^{\prime }-{\overset{ˆ}{E}}_{1}^{\prime }|\rangle_{x,y,f}\right\|}_{2}}{{\left\|\langle |{\overset{ˆ}{E}}_{1}^{\prime }|\rangle_{x,y,f}\right\|}_{2}},$$

(28b) $$\epsilon_{J_{2}^{\prime }}=\frac{{\left\|\langle |J_{2}^{\prime }-{\overset{ˆ}{J}}_{2}^{\prime }|\rangle_{x,y,f}\right\|}_{2}}{{\left\|\langle |{\overset{ˆ}{J}}_{2}^{\prime }|\rangle_{x,y,f}\right\|}_{2}}.$$

A summary of the condition numbers, the relative errors of the auxiliary matrices A and B, and the relative errors of the calibration parameters $E_{1}^{\prime }$ and $J_{2}^{\prime }$, are given in Table I. It is evident that the equidistant angles over π period have an overall better performance compared to the two sets of angles that minimize $\kappa_{P}$. The error of calibrated $E_{1}^{\prime }$ and $J_{2}^{\prime }$ in the case of choosing matrix formulation B, leading to 3 equidistant angles with $\Delta \phi =\frac{\pi }{3}$ are approximately 7% and 10%, respectively. These values are much higher for the angle minimization $\kappa_{P}$ when choosing the matrix formulation A. This indicates the advantage of using the relationship (17) that its minimized condition number suggests equidistant angle sampling over a period π, compared to (13) that results in nontrivial sets of angles.

## IV. Polarimetric Sample Characterization

In the previous section, we described complete calibration of the polarimetric scanner with a non-ideal polarizer. In this section, we demonstrate characterization of birefringent samples by extracting the Jones matrix from the following equation that described the measurement system, given by

(29) $$E_{\text{M}}=J_{2}^{\prime }R\left(-\phi_{1}\right)J_{\text{S}}R\left(\phi_{1}\right)E_{1}^{\prime },$$

where $\phi_{1}$ is the rotation angle of the sample. Applying a left multiplication by $J_{2}^{\prime (-1)}$ and $R{\left(-\phi_{1}\right)}^{\left(-1\right)}$ to (29) results in

(30a) $$E_{2}^{\prime (r)}\left(\phi_{1}\right)=J_{\text{S}}E_{1}^{\prime (r)}\left(\phi_{1}\right),$$

where the superscript (r) indicates the rotation of the Jones vectors to match the coordinate system of the sample (note that $R{\left(-\phi_{1}\right)}^{-1}=R\left(\phi_{1}\right)$), and therefore

(30b) $$E_{1}^{\prime (r)}\left(\phi_{1}\right)=\left[\begin{array}{c} E_{1,x}^{\prime (r)}\left(\phi_{1}\right)\\ E_{1,y}^{\prime (r)}\left(\phi_{1}\right) \end{array}\right]=R\left(\phi_{1}\right)E_{1}^{\prime },$$

and $E_{2}^{\prime (r)}$ is the reflected and rotated electric field from the sample and is given by,

(30c) $$E_{2}^{\prime (r)}\left(\phi_{1}\right)=\left[\begin{array}{c} E_{2,x}^{\prime (r)}\left(\phi_{1}\right)\\ E_{2,y}^{\prime (r)}\left(\phi_{1}\right) \end{array}\right]=R\left(\phi_{1}\right)J_{2}^{\prime -1}E_{\text{M}}.$$

Since (30a) is under-determined, at least two sets of measurements with different and independent rotation angles $\phi_{1}$ and $\phi_{2}$ of the sample should be concatenated to extract $J_{\text{S}}$ resulting in

(31) $$J_{\text{S}}=\left[\begin{array}{cc} E_{2,x}^{\prime (r)}\left(\phi_{1}\right) & E_{2,x}^{\prime (r)}\left(\phi_{2}\right)\\ E_{2,y}^{\prime (r)}\left(\phi_{1}\right) & E_{2,y}^{\prime (r)}\left(\phi_{2}\right) \end{array}\right]{\left[\begin{array}{cc} E_{1,x}^{\prime (r)}\left(\phi_{1}\right) & E_{1,x}^{\prime (r)}\left(\phi_{2}\right)\\ E_{1,y}^{\prime (r)}\left(\phi_{1}\right) & E_{1,y}^{\prime (r)}\left(\phi_{2}\right) \end{array}\right]}^{-1}.$$

As a spatially uniform test sample an x-cut lithium niobate LiNbO_3_ crystal wafer was chosen, due to its large birefringence in the THz regime [64], [65]. Reflection measurements were recorded at various angels, and only the first reflected pulse from the air-crystal interface is used in the analysis. This assumption preserves the generality of the method for thick or highly absorptive samples. The Jones matrix of the sample can be described as

(32a) $$J_{\text{S}}=R\left(-\phi_{0}\right)J_{\text{S}0}R\left(\phi_{0}\right),$$

(32b) $$\begin{array}{c} J_{\text{S}0}=\left[\begin{array}{cc} r_{o} & 0\\ 0 & r_{e} \end{array}\right], \end{array}$$

where $r_{o}$ and $r_{e}$ are the reflection coefficients along the ordinary and extraordinary axes of the crystal, and $\phi_{0}$ is the unknown orientation angle of its principal axes. Since the rotation matrix R is unitary (i.e., $R(-\phi )=R(\phi {)}^{T}=R(\phi {)}^{-1}$), the Jones matrix $J_{\text{S}}$ extracted from (31) is related to $J_{\text{S}0}$ by a unitary similarity transformation and therefore shares the same eigenvalues. Because $J_{\text{S}0}$ is diagonal in the crystal principalaxis frame, its eigenvalues are equal to the intrinsic reflection coefficients $r_{o}$ and $r_{e}$. Consequently, the eigendecomposition of $J_{\text{S}}$ directly results in the intrinsic reflection coefficients of the crystal, independent of the orientation of its principal axes [66], [67].

**Fig. 7.**
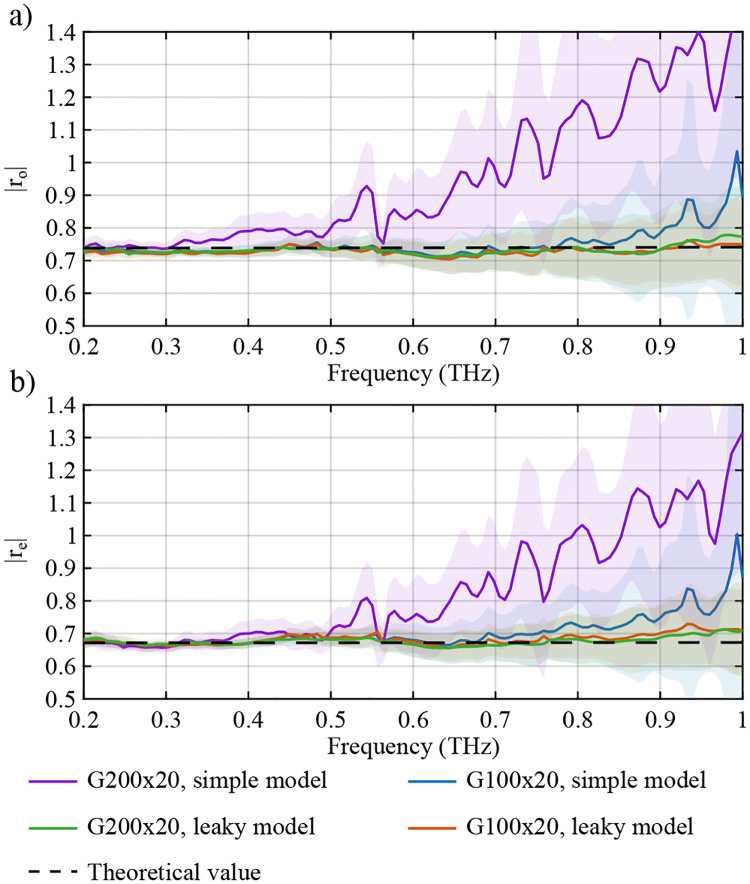
Reflection coefficient of air and x-cut LiNbO_3_ along the crystal’s (a) o-axis and (b) e-axis extracted using the calibration obtained with the simple (purple) and the leaky (green) models of G200×20, and the calibration with the simple (blue) and the leaky (red) models of G100×20 polarizer. The theoretical value of the reflection coefficients along the two crystal axes are shown with black dashed lines. The solid lines and shaded area indicate the averages and standard deviations of the spectra, respectively, over a 6 mm×6 mm area in the center of the FOV. The Jones matrices are calculated using −30° and 60° angles.

Four calibration scenarios were compared using the two different polarizers G100×20 and G200×20, modeled by the simple or leaky polarizer equations in (4) and (6), respectively. The eigenvalues of the Jones matrix of the crystal $J_{\text{S}}$ were calculated using (31) for each of the calibration cases. Figs. 7(a) and (b) show the amplitude of the reflection coefficients of the crystal along its o- and e-axes, respectively. Importantly, the two calibration sets associated with the simple model of the polarizer result in a systematic error between the extracted and theoretical reflection coefficients. Because the leakage of the WGP increases with decreasing wavelength, the assumption underlying the simple polarizer model is not valid at higher THz frequencies. As a result, the extracted $r_{o}$ and $r_{e}$ increasingly deviate from their theoretical predictions. Furthermore, since the leakage of G200×20 is greater than that of G100×20, the corresponding estimates of $J_{\text{S}0}$ obtained using the simple model show larger deviations for G200×20. However, when the calibration parameters incorporate the leaky polarizer model, the extracted reflection coefficients agree closely with theoretical predictions [65]. This indicates that using the proposed calibration framework the sample characterization is effectively independent of the leakage of the calibrating polarizer.

**Fig. 8.**
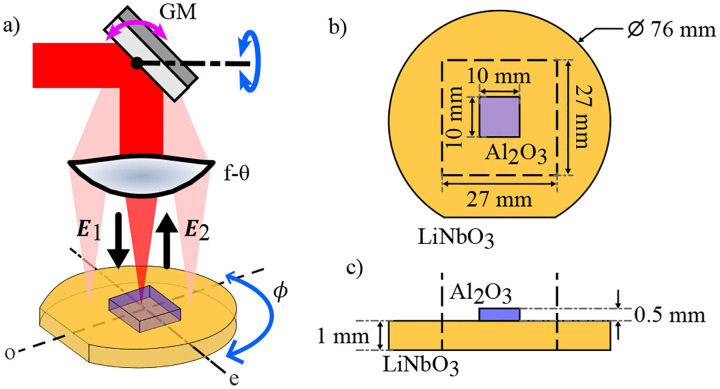
a) Schematic of scanning of a rotating stack of crystals. The ordinary and extraordinary axes of the crystal are shown with o and e. (b) Top view and (c) cross-section of the stack of lithium niobate LiNbO_3_ and sapphire Al_2_O_3_ crystals. The dashed lines indicate the approximate FOV of the system.

**Fig. 9.**
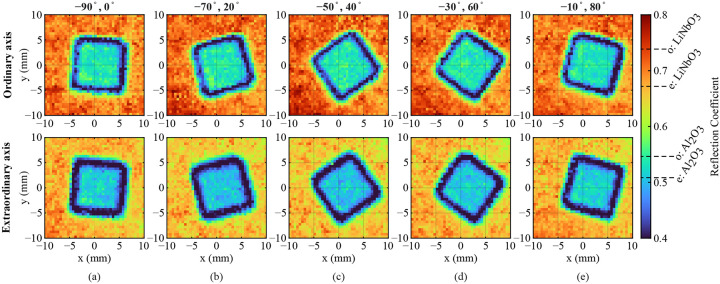
The maps of the reflection coefficients of Al_2_O_3_ (square crystal in the center) and LiNbO_3_ (background) obtained from the eigendecomposition of $J_{\text{S}}$. The read-out rotation of the mount - close to $\phi_{0}$ - for the two combined measurements are shown on the top of each pair of the Jones matrix maps. The predicted pseudo-color of the two crystals are shown separately on the colorbar. The useful FOV is limited due to the shift of the secondary measurement to compensate for the non-central pivot point.

The above validation experiment using a uniform LiNbO_3_ wafer provided a procedure to compare the spectroscopic results obtained using different calibration sets. However, practical applications require spatially resolved characterization. In the following, the proposed method is applied to a stack of two birefringent crystals. A 1 cm × 1 cm sapphire crystal (Al_2_O_3_) with a thickness of 0.5 cm is placed on the top of the LiNbO_3_ wafer, as shown in Fig. 8. Note that since the calibration parameters are spatially dependent, the rotation of the samples to obtain the two necessary measurements requires an accurate mapping of the corresponding pixels. Measurements at various angles between −90° and 90° are recorded. $E_{2}^{\prime (r)}$ and $E_{1}^{\prime (r)}$ are formed as follows

(33a) $$E_{1}^{\prime (r)}=\left[\begin{array}{cc} E_{1,x}^{\prime (r)}\left(\phi_{1},x,y\right) & E_{1,x}^{\prime (r)}\left(\phi_{2},u,v\right)\\ E_{1,y}^{\prime (r)}\left(\phi_{1},x,y\right) & E_{1,y}^{\prime (r)}\left(\phi_{2},u,v\right) \end{array}\right],$$

(33b) $$E_{2}^{\prime (r)}=\left[\begin{array}{cc} E_{2,x}^{\prime (r)}\left(\phi_{1},x,y\right) & E_{2,x}^{\prime (r)}\left(\phi_{2},u,v\right)\\ E_{2,y}^{\prime (r)}\left(\phi_{1},x,y\right) & E_{2,y}^{\prime (r)}\left(\phi_{2},u,v\right) \end{array}\right],$$

(33c) $$\begin{array}{c} \left[\begin{array}{l} u\\ v \end{array}\right]=R\left(\phi_{1}-\phi_{2}\right)\left(\left[\begin{array}{l} x-x_{0}\\ y-y_{0} \end{array}\right]\right)+\left[\begin{array}{l} x_{0}\\ y_{0} \end{array}\right], \end{array}$$

where (x,y) denotes the coordinate in the first measurement, (u,v) represents the corresponding pixel coordinate from the second measurement mapped onto the first, and $\left(x_{0},y_{0}\right)$ is the pivot point of the rotation. $J_{\text{SO}}$ is obtained from the eigendecomposition of (31). For simplicity of the coordinate transformation, angle pairs 90° apart were selected. In addition, selecting 90° apart pair of angles ensures a well-conditioned equation. Since the eigenvalues of $J_{\text{S}}$ are the reflection coefficients, in all cases $\phi_{1}$ and $\phi_{2}$ were assumed to be 0° and 90°, and the principal orientation of the crystals $\phi_{0}$ was not inserted into the equations. Fig. 9 shows the maps of $J_{\text{S}}$ with various pair of angles. Since the reflection coefficients of the two crystals are relatively constant over the bandwidth, the absolute values of the reflection coefficients are averaged between 0.3–0.6 THz, and are shown by the pseudo-color map. The proposed method can accurately map the predicted values of the reflection coefficients of the two crystals along their two optical axes [65], [68], [69], independently of the orientation of the principal axes of the crystals.

## V. Discussion

This study presents an *in situ* technique for the calibration of the THz polarimetric PHASR Scanner using a rotating wire-grid polarizer (WGP) in front of a mirror. However, free-standing wire-grid polarizers have a limited useful bandwidth determined by the diameter and period of the wires, which is on the order of tens of micrometers. Therefore, the extinction ratio of these polarizers is frequency dependent and is not ideal in the THz regime [53]. The frequency response of the polarizer leakage is defined as a complex parameter ηexpiδ, where η and δ are the extinction coefficient and phase retardance of the polarization parallel to the orientation of the wires [57]. As an approximation, it is shown in the supplementary material that neglecting this leakage causes a systematic relative error of the measured electric field proportional to $\eta^{2}$. Therefore, reliable calibration of the polarimetric scanner depends on the accurate characterization of the WGP. Separate transmission measurements of the calibrating polarizer were performed to characterize its frequency-dependent extinction ratio. Simultaneous characterization of the polarizer along the calibration of the system is also feasible using optimization techniques [60]. Using this leaky model of the WGP in the Jones matrix equations ensures that the calibration of the system is insensitive to the imperfect response of the polarizer.

The calibration technique using the rotating WGP lead to two equivalent system of equations that link the measured electric field to the calibration parameters via angle dependent matrices. These two matrices were compared to find a robust calibration framework with minimum number of necessary measurements by analyzing their condition numbers. The new equation system is formulated using orthogonal harmonic bases, which can also be viewed as an analogues Fourier series interpretation, and suggests that the rotation angles of the calibrating polarizer should be circularly equidistant with a period of π to minimize sensitivity to error propagation. We show that at least 3 rotation angles of the polarizer are required to determine the 5 calibration parameters in the Jones matrix $J_{2}^{\prime }$ and the Jones vector of the incident electric field $E_{1}^{\prime }$. Various sets of angles satisfying the minimum condition number of the two equations matrices were compared against a calibration measurement containing 18 angles. The relative error of $J_{2}^{\prime }$ and $E_{1}^{\prime }$ calibrated using only 3 optimized angles remains less than 10% compared to the full set of 18 angles.

After careful and robust calibration of the system, we derived a framework for extracting the Jones matrix of a sample based on eigendecomposition of the concatenated matrix of measurements. The lithium niobate LiNbO_3_ crystal was used as a birefringent test sample. The reflection coefficients of LiNbO_3_ along its two optical axes were obtained from the eigendecomposition of the extracted Jones matrix $J_{\text{S}}$, which accounts for the unknown orientation of the crystal. The calculated reflection coefficients were compared with the theoretical values to confirm the robustness of the technique. It was shown that the Jones matrix of the crystal extracted from a calibration set that neglects the leakage of the polarizer results in a systematic error. Furthermore, the Jones matrix of a stack of sapphire crystal on top of the lithium niobate wafer was mapped to demonstrate the imaging capability of the system.

The PCAs integrated into the PHASR Scanner perform THz acquisition using the electronically controlled optical sampling (ECOPS) technique [56], which enables several kHz scan rate leading to a rapid calibration procedure - each THz image at a certain WGP rotation angle is acquired in less than 1 minute. Although the system characteristics remain mostly unchanged over time, this fast calibration framework with a minimal number of calibration measurements is particularly advantageous for frequent calibration. However, ECOPS introduces additional experimental considerations. The pulse jitter and drift arising from the non-ideal femtosecond laser repetition rate variations can introduce a substantial source of error. This error is explicitly accounted for in the calibration procedure and is discussed in Section I of the Supplementary Material.

In future studies, we will present applications of the polarimetric PHASR Scanner to map the Jones matrix of various polarization-sensitive samples with anisotropic structures, birefringence and dichroism signatures, and scattering medium. In addition, methods will be provided to describe the full decomposition of the Muller matrix of samples [70].

## VI. Conclusion

We present a robust and rapid calibration of the THz polarimetric PHASR Scanner using a rotating wire-grid polarizer in front of a mirror. The non-ideal frequency-dependent response of the calibrating polarizer gives rise to a systematic error in the calibration of the polarimetric scanner, and therefore, a leaky model is used to compensate for this error. Moreover, a mathematical framework is presented to find the optimal rotation angles of the calibrating polarizer. Using the improved calibration method, the Jones matrices of two birefringent crystals are mapped that closely match with their theoretical values using eigendecomposition of the new measurement matrix.

## Figures and Tables

**TABLE I:** Summary of the relative error of A, B, and the relative error of calibration parameters $E_{1}^{\prime }$ and $J_{2}^{\prime }$ using various sets of angles, along with their condition number.

	Random	Minimizing $\kappa_{P}$		Minimizing $\kappa_{Q}$		
Angle set	10°, 30°, 110°	23°, 67°, 135°	157°, 113°, 45°	0°, 60°, 120°	20°, 80°, 140°	40°, 100°, 160°
$\kappa_{P}$	4.88	1.777	1.777	2	2	2
$\kappa_{Q}$	4.014	1.812	1.812	1.4142	1.4142	1.4142
$\epsilon_{A}$	22.44%	13.54%	12.73%	10.24%	9.77%	11.06%
$\epsilon_{B}$	19.35%	13.6%	11.6%	9.49%	9.57%	9.73%
$\epsilon_{E_{1}^{\prime }}$	13.56%	11.66%	7.17%	6.27%	7.82%	6.94%
$\epsilon_{J_{2}^{\prime }}$	19.77%	19.50%	13.15%	9.91%	12.76%	10.97%
